# Shaping nature outcomes in corporate settings

**DOI:** 10.1098/rstb.2022.0325

**Published:** 2024-06-10

**Authors:** Jan Bebbington, Robert Blasiak, Carlos Larrinaga, Shona Russell, Madlen Sobkowiak, Jean-Baptiste Jouffray, Henrik Österblom

**Affiliations:** ^1^ Pentland Centre for Sustainability in Business, University of Lancaster, LA1 4YW, UK; ^2^ Stockholm Resilience Centre, Stockholm University, 106 91 Stockholm, Sweden; ^3^ Departamento de Economía y Administración de Empresas, Universidad de Burgos, 09001 Burgos, Castilla y León, Spain; ^4^ Department of Management, University of St Andrews Business School, St Andrews, KY16 9AJ, UK; ^5^ EDHEC Business School, 59046 Lille, France; ^6^ Stanford Center for Ocean Solutions, Stanford University, Stanford, CA 94305, USA; ^7^ Anthropocene Laboratory, Royal Swedish Academy of Sciences, SE-104 05 Stockholm, Sweden

**Keywords:** company decision-making, biodiversity accounting, information governance

## Abstract

Transnational companies have substantive impacts on nature: a hallmark of living in the Anthropocene. Understanding these impacts through company provision of information is a precursor to holding them accountable for nature outcomes. The effect of increasing disclosures (of varying quality) is predicated on ‘information governance’, an approach that uses disclosure requirements to drive company behaviour. However, its efficacy is not guaranteed. We argue that three conditions are required before disclosures have the possibility to shape nature outcomes, namely: (1) radical traceability that links company actions to outcomes in particular settings; (2) developing organizational routines, tools and approaches that translate strategic intent to on-the-ground behaviour; and (3) mobilizing and aligning financial actors with corporate nature ambitions. While disclosure is key to each of these conditions, its limits must be taken into account and it must be nested in governance approaches that shape action, not just reporting.

This article is part of the theme issue ‘Bringing nature into decision-making’.

## 1. Introduction

This paper focuses on how transnational companies [1] attempt to incorporate nature in their decision-making processes and, especially, how the outcomes of these decisions can be reflected in company accounts of performance. Accounts of corporate biodiversity impacts serve many purposes. In the first instance, disclosure of actions, rationales and the outcomes from actions provides information to those affected by companies so that they might hold them to account. This includes stakeholders being able to seek redress for harms as well as being able to influence future activities so that harm is avoided in the first place. Second, accounts of nature might themselves be inputs into the decision-making processes of company funders (such as banks) and owners, to the extent to which these actors value nature. These financial stakeholders possess the power to influence corporate actions by virtue of their close economic relationships with company managers. Within this framing, there are three critical success factors that need to be satisfied before transparency can enhance corporate responsibility for nature outcomes. First, there must be a robust way to link nature outcomes (in a particular location) with company decisions making activities: these two aspects might be physically distant from each other. The provision of information that delineates these links, based on traceability, is a pre-condition and enabler of company transparency and decision-making. Second, there is a need to translate company strategic intentions with respect to nature outcomes into operational routines that inform actions taken on the ground at a particular location: tracing the effects of these decisions and being transparent about actions and effects are also required. Third, financial actors who themselves have a varying propensity to take nature into account in their decisions must create the right incentives in support of company decision-making. A common way to align incentives between companies and external actors is through corporate transparency about performance.

While there is a growing momentum for nature-related disclosures, it is important to note that reliance on corporate transparency is both the outcome of market failure as well as an example of a particular mode of governance. If there were effective nature governance in all locations where companies operate then we might still expect disclosures to be made on the degree of compliance with laws and regulations, and this would provide evidence that corporate actions aligned with ecologically sound outcomes. This situation does not pertain, with nature governance being uneven across the globe. Unequal strength of regulation matters because companies operate globally. Indeed, globalization and the rise of transnational companies mean that companies that are headquartered in one country have impacts across many others, and they face a complex patchwork of regulation of varying strength [2]. State sovereignty means that a country can only directly regulate company actions where those actions take place or in their country of incorporation (that is, where the company is headquartered). Governance, however, can still have effects beyond a particular country through, for example, import bans on products into a country (e.g. the EU draft law on banning six core agricultural commodities—beef, soya, palm oil, coffee, cocoa and timber—from entering the single market if they are associated with deforestation). In addition, an EU Directive is under negotiation that will require companies to demonstrate that due diligence (including from an environmental perspective) has been undertaken along value chains, thereby providing insight into effects that are outside of the EU. Despite these examples of regulation with cross-border effects, the general point holds: that no one country can fully regulate a company's nature impacts because some of them will take place or have effects outside its jurisdiction.

The inherent difficultly of governance highlighted above, along with less political appetite for command and control approaches to regulation, have resulted in the rise of mandated information disclosure as a way of regulating behaviour: this approach is termed ‘information governance’ [3] or ‘targeted transparency’ [4]. However, providing information about performance is not sufficient to support nature-positive outcomes. Rather, disclosures must be placed within wider governance processes where state and private actors require or influence companies to reduce impacts upon, and ultimately to restore, nature. Indeed, transparency is a necessary but far from sufficient basis for corporate accountability [5–7].

This focus on corporate transparency requirements, as distinct from the regulation of actions and impacts, forms the basis of this contribution to the special issue on ‘Bringing nature into decision-making’. In §2, we introduce the nature of companies, the decisions they make and how they seek to discharge accountability to share- and stakeholders. We use an organizational science-based understanding of when, how and under what conditions companies (along with their financial stakeholders) can take nature into account. This literature is unlikely to be familiar to readers of this journal. As a result, one contribution of this paper is to foster an inter-disciplinary understanding of governance that focuses on company nature disclosures alongside the more familiar natural resource governance (e.g. regulatory approaches that may specify areas to be protected from company activity, resources utilization or pollution limits). Section 3 returns to the examination of how corporate decision-making can be effectively linked to nature outcomes, addressing the three critical success factors outlined in the opening. Section 4 provides examples from practice for each of the factors, noting that these practices are not yet widespread or mainstream. Rather, the examples will need to be further developed and diffused across sectors and countries if we are to see companies acting as stewards of the biosphere [8], an idea that is itself contested [9], and whether transparency is an enabler of this ambition [10]. Finally, some concluding comments are made.

## 2. Company decision-making through transparency

While companies are characterized in diverse ways, they are often described in a narrow sense as ‘externalizing machines' that are unable to exercise agency in service of goals beyond their short-term economic self-interest [11,12]. In contrast, there is a stream of work that takes a more circumspect view, and which recognizes that companies are subject to a myriad of pressures to take wider social and ecological matters into account and are willing and able to do so when system conditions are conducive to change [13,14]. This is increasingly the case as company managers and owners are starting to recognize that they are actively shaping outcomes in the Anthropocene [1,14,15]. Beliefs about how much change is required differ, as reflected in adaptive and transformative governance approaches [16,17].

Adaptive governance assumes that companies need to respond to novel socio-ecological assemblages through collective action and coordination in order to allow businesses to draw down and transform resources. Transformative governance assumes that systemic shifts are necessary for corporate and societal functioning [18,19]. To evaluate the extent to which, and how, companies might support adaptive (and perhaps ultimately transformative) approaches, it is essential to distinguish two layers of governing activity: governance focused on natural systems and governance focused on information provision about the outcomes of company interactions with the natural system.

Governance of the natural system in which companies operate might include requirements for environmental quality outcomes to be attained, resource extraction limits and/or proscription against certain activities. Here company decision-making should focus on ensuring that actions comply with environmental standards. At the same time, companies are undertaking additional decision-making in order to understand what kinds of actions they should be taking that are driven by their own needs (e.g. to ensure a flow of materials from an ecosystem that they are dependent upon). These kinds of decisions might also be enacted on a collective basis, such as through a voluntary environmental programme [20].

The second broad category of governance focuses on information that articulates the outcomes of company decision-making. Here there are links between internal decision-making and this externally focused information because one pre-supposes the other. This is why, in the absence of being able to directly observe company decision-making, there is a focus on information provision. This information will often describe both the processes that informed the decision as well as the outcomes of decisions. Such transparency is how a company makes itself comprehensible to others [17] and uses the perceived power of shame and the threat of effective stakeholder pressure to drive behaviour [21]. In this way, transparency underpins the possibility for accountability: where someone can affect the company given the information that has been provided [5]. In addition, information governance assumes that company disclosures shape and reshape economic processes with a follow-on effect on nature outcomes [22]. The impossibility of regulating all company action for biosphere stewardship means that (despite its limitations) information governance plays a critical role in company–nature intersections [2,3,12].

An information governance approach is evident in Target 15 of the Kunming–Montreal Global Biodiversity Framework (see https://www.cbd.int/article/cop15-final-text-kunming-montreal-gbf-221222), which asks countries to take ‘legal, administrative or policy measures to encourage and enable business, and in particular to ensure that *large and transnational companies and financial institutions*: (a) Regularly monitor, assess, and transparently disclose their risks, dependencies and impacts on biodiversity, including with requirements for all large as well as transnational companies and financial institutions along their operations, supply and value chains and portfolios’ (emphasis added). How this intention will play out in practice is yet to be determined, but it is certainly likely to utilize the disclosure requirements outlined in table 1. This aspiration contrasts with the language from the 2010 Aichi Target Four (see https://www.cbd.int/sp/targets), which simply contained a general exhortation for business to have implemented plans for sustainable production and consumption and to keep nature impacts within safe ecological limits. The newer requirements provide more detail of what actions should be undertaken (e.g. identify risks, dependencies and impacts) and are in line with due diligence approaches that incorporate own activities and those along value chains [26,27]. Such requirements reflect the kinds of decisions companies are making with respect to biodiversity, for example, recognizing that companies depend upon nature because it is the source of raw materials and also buffers company direct and indirect impacts. How corporate action might be enabled in this context rests on three elements (see §3).

**Table 1.** Examples of future nature-related disclosure requirements

The Global Reporting Initiative (GRI) is a standard-setting body that describes what appropriate corporate accounts of impacts would entail. Their suite of standards includes core information requirements (e.g. identifying impacts that are material to a company), topic standards (on economic, environmental and social aspects) and sector standards (that draw together and customise topic standards for particular sectors).
The GRI launched a revamped biodiversity topic standard (GRI 304) for public consultation in December 2022 [23,24] and they identified the following topics as being necessary for creating a robust company account of biodiversity interactions (not all these elements are likely to survive public consultation, with the new standard expected to be released in 2024): • Disclosure 304-1: information about the operational sites of the organization and its suppliers that cause or contribute to the most significant actual and potential impacts on biodiversity. These sites are to be geolocated and their size provided along with how close they are to legally protected, internationally recognized areas and other areas of high biodiversity value. Subsequent disclosures are required for these operational sites. • Disclosure 304-2: information that will provide an understanding of the activities responsible for the direct drivers of biodiversity loss. This includes greenhouse gas emissions; invasive alien species; land and sea use change; ecosystem conversion; overexploitation of resources and pollution. Disclosure is also sought regarding processes used to monitor the direct drivers of biodiversity loss throughout company activities and along supply chains. • Disclosure 304-3: information on the state of biodiversity for each site identified by ecosystem type, size and condition (for a baseline year and the current reporting period). This includes information on species names and extinction risks. • Disclosure 304-4: information on significant ecosystem services and beneficiaries that are or could be affected by the organization and their suppliers. These include provisioning, regulating and maintenance, and cultural services. • Disclosure 304-5: information on the actions taken to manage the organization's direct drivers of biodiversity loss and its impacts on the state of biodiversity and ecosystem services. This includes how a company uses the mitigation hierarchy (actions to avoid, minimize, restore and offset impacts) along with the percentage of sites with management plans and a description of how actions are implemented. • Disclosure 304-6: requires information on policies and commitments to halt and reverse the loss of biodiversity (in line with the 2050 Goals and 2030 Targets in the Convention on Biological Diversity's post-2020 Global Biodiversity Framework). The scope of these policies in terms of a company's own activities, its supplier and downstream companies are sought along with any goals, targets, base year and indicators used (with information on how these measures are determined). • Disclosure 304-7: information on how the organization respects national legal requirements to achieve the fair and equitable sharing of benefits arising from utilizing genetic resources and associated traditional knowledge.
The EU Council approved the Corporate Sustainability Reporting Directive (CSRD) on 28 November 2022, thereby setting in motion a process for EU member states to enact its requirements in their national laws (they have 18 months to do this). The CSRD creates an additional sustainability reporting requirement (there is an existing EU reporting requirement in the form of the Non-Financial Reporting Directive) and expands the number of EU and non-EU companies subject to reporting requirements. Critically, disclosure will be required on the due diligence processes implemented by a company in relation to sustainability matters and the actual and potential adverse sustainability impacts of in-scope company's operations and value chain. It is likely that these requirements will become binding for financial years starting in 2024. In parallel with the development of this regulation, the European Financial Reporting Advisory Group has created proposed reporting standards to provide technical details on reporting requirements. The European Sustainability Reporting Standard, E4 [25] describes the type of data that would be required to discharge accountability for biodiversity and ecosystems impacts as well as requiring disclosures of how policies are developed and implemented for nature decision making, metrics that track biodiversity interactions, targets and action plans.

## 3. Elements to support companies and nature decision-making

As outlined in the opening of the paper, there are three critical success factors for establishing company–nature links, themselves a precursor for corporate accountability. Each of these is further elaborated below.

### (a) Linking companies and ecosystems

If a company operated within a single ecosystem (in the ecological sense of the word) that was itself subject to governance that is based on sound science, the decisions that would have to be made with respect to nature would be straightforward, albeit with a necessity to spatially locate specific operations in order to understand ecosystem dependencies and impacts. Specifically, a company in this setting would have to make operational decisions that resulted in them adhering to legal requirements (noting that even with good governance, externalities may still exist given that all ecosystems are interconnected). Except for smaller organizations with limited geographic range, these conditions do not pertain, especially not for transnational companies that operate in more than one country [1]. As such, there is innate complexity for a company to make decisions about nature and provide information on their performance because of this dispersion of impact versus control exercised by them.

Further, central to conventional organizational accounts is the entity concept, which dictates that only the costs borne by organization (i.e. the entity) are of relevance, thereby obscuring the interplay between the organization and nature [28]. As a result, there is a mismatch between the legal abstraction of the organization and the actual physical and biological flows produced by its activity. Thus, the very act of attempting to create an organizational-level nature account is ecologically reductionist and not the same as accounting for an ecosystem (most obviously, company accounts cannot easily allow cumulative impacts to be identified).

While it is ecologically robust for nature impacts to be evaluated at the ecosystem level [29–31], companies remain major drivers of nature outcomes, either directly or through their value chains. Therefore, company-level nature disclosure is necessary, despite the complexity of this endeavor [8,15], the relative paucity of disclosure to date [24] and the lack of ambition evident in current disclosures [32]. It is also the case that advances in ‘big-data’ platforms (such as the Global Fishing Watch (see https://globalfishingwatch.org/) and Global Forest Watch (https://www.globalforestwatch.org/)) means that if companies do not undertake this linking work, others will do so. This creates the potential for companies to be held to account regardless of whether they publish data, and it creates incentives for companies to produce their own data rather than having to respond to exposés created by third parties.

Finally, questions about the capacity of investors and other stakeholders to understand the complexity and attend to both corporate- and ecosystem-level disclosure at the same time require both ecological and organizational literacy, which would require a substantive ‘step-up’ in understanding.

This begs the question of what information would discharge accountability or shed light on the decision-making processes that companies undertake. Standards are developing in this area that will have effects beyond national settings (table 1). For instance, the Global Reporting Initiative (GRI) is a framework increasingly used by companies across the world. Likewise, EU regulations will have an effect beyond the EU as the standards apply to non-EU companies that generate annual net turnover of €150 million in the EU and have at least one subsidiary or branch in the EU. In this way, the EU-based regulation will have extra-territorial effects. Moreover, the existing and emerging information requirements will require considerable ecological literacy by organizations.

A critical determinant of what information should be provided is the conception of materiality adopted in standards and company reporting approaches. Materiality refers to how you decide if a piece of information ‘matters’, and it comes in two forms. *Impact materiality* refers to the impact of corporate actions on the biosphere, whereas *financial materiality* depends on the extent to which nature impacts on a company's financial position. These are distinctive approaches, with the GRI using impact materiality while the EU Corporate Sustainability Reporting Directive (CSRD; see https://eur-lex.europa.eu/legal-content/EN/TXT/?uri=CELEX:32022L2464) uses both impact and financial materiality (often called double materiality). More traditional accounting standard setters (who are also moving into non-financial reporting regulation) focus on financial materiality. An extended discussion of these distinctions is beyond the scope of this paper but they radically affect the kinds of information that might be provided.

### (b) Translating aspirations into operations

While linking companies with ecosystems provides knowledge of the significance of corporate actions from an ecosystem perspective, the next critical success factor is linking knowledge and action [14]. Even if companies are well-informed about their impacts on nature, translating company strategies to reduce impacts and restore nature into operational targets and control mechanisms is difficult [33]. For example, the so-called *Blue Acceleration* [34] is taking place despite the availability of increasingly complex information about ocean impacts and an understanding of who is operating in the marine environment [35]. Evidence that knowledge *per se* does not mobilize action begs the question of what kind of information is likely to bring nature into company decision-making.

In this regard, organizational accounts not only need to be provided but also need to function as mediating instruments [36], mobilizing and multiplying ideas across companies (e.g. about the need for biosphere stewardship) and translating ambitions into specific metrics that, once embedded in companies, create visibilities and routines that focus the attention of company managers. This process must happen despite the problematic character of nature accounts (due to the entity concept previously highlighted [28–31]). From an internal company perspective, there may be benefits from developing (imperfect) accounts because they create insight into nature interactions that may initiate internal discussion, risk evaluations and action planning. Understanding how this happens inside companies and the potential for positive spillover effects in terms of company capability and learning remains in its infancy [37,38].

### (c) Shaping financial system responsiveness

The final element in this section relates to identifying how financial system actors can enable company actions. Company owners and those who fund companies are the most powerful financial actors in this context [39] and it has only recently been identified that financial stability relies on well-functioning ecosystems [40], mirroring recent realizations that climate stability is also critical for financial system stability. New coalitions have developed to support this process and contribute to the environment and climate risk management in the financial sector [41].

Once again, information governance is being used to draw investor attention to nature impacts, mirroring more developed interventions for climate-related impacts. An example of such a mechanism (which is being copied in other jurisdictions) is the EU's Sustainable Finance Disclosure Regulation (SFDR): (EU) 2019/2088 (https://eur-lex.europa.eu/eli/reg/2019/2088/oj). The SFDR focuses on banks, insurers, asset managers and investment firms that operate in the EU and it requires them to provide information about how they address sustainability risks. In addition, and potentially more powerfully, there are also ‘product’ level disclosure requirements, where financial products (such as investment funds) claiming to have positive environmental impacts are required to demonstrate how such an impact arises. Substantive rules about what is classified as ‘environmentally sustainable’ also exist in the form of the EU Taxonomy (https://eur-lex.europa.eu/legal-content/EN/TXT/?uri=CELEX:32020R0852). This regulation was devised to address ‘greenwashing’ in the investments sector, as well as to redirect capital flows into sustainable activities to support the EU's Action Plan on Financing Sustainable Growth (https://eur-lex.europa.eu/legal-content/EN/TXT/?uri=CELEX:52018DC0097).

Regardless of this additional layer of disclosure requirements, a more generalized appreciation of the environmental risks to financial stability and financial returns, as well as the dependency of companies and the financial system on well-functioning ecosystems, continues to prompt institutional innovation. For example, rating of investments against ‘environmental, social and governance’ (ESG) criteria is gaining momentum, although the robustness of these criteria and their effect on company performance are open questions, with conflicting academic findings that are differentially interpreted depending upon subject framing and the investment context [42]. Another mediating instrument is the Taskforce on Nature-related Financial Disclosures (https://tnfd.global/), once again mirroring the more longstanding and (in some places compulsory) disclosures on Climate-related Financial Disclosures. These disclosure requirements encourage companies, as account providers, to articulate how climate/nature risks will affect financial returns and to provide accounts of company impacts on biodiversity. This may, if considered holistically, provide evidence for why a company and its owners or lenders might include such matters in their decision-making.

## 4. Examples of nature decision-making

Table 2 presents examples of nature-based reporting that draw on the previously identified critical success factors for establishing robust company–nature links: that is, linking companies and ecosystems, translating aspirations into operations and shaping financial system actors. It must be stressed that these emerging examples do not represent widespread practice.

**Table 2.** Examples of company action to shape nature outcomes.

***Linking companies and ecosystems***
Cargill is a supplier of feeds for the food sector and, in their *Soy in South America* report [43], they trace the soy that they produce and purchase through their supply chain with locations in South American countries. These locations are geospatially located [44] with data on the degree of deforestation in each polygon obtained from satellite images and country-level data sources. In this respect, traceability and the possibilities offered by Global Information System-based analysis create the possibility for nature accounts.
Nippon Suisan Kaisha, Ltd. (Nissui), is involved in wild capture fisheries and aquaculture [45], and it provides data on the sustainable use of marine resources that combine information on the volume and species harvested from the ocean (using traceability data) to evaluate the sustainability credentials of the fisheries from which these raw materials were obtained. They use a methodology developed by the Ocean Disclosure Project (https://oceandisclosureproject.org/), which contains information on the sustainability of fish populations, to link (mediate) between their raw materials and the sustainable status of the ecosystem. The outcome of this analysis also highlights data gaps, as well as species and fisheries where improvement could be sought by the company itself (for example, through actions taken to translate strategy to operations). In this case, the linking of impacts to locations is a precursor to taking action and demonstrates the interdependence between these factors.
***Translating aspirations into operations***
Cermaq, an aquaculture company [46] translates aspirations to operations by collecting detailed information about non-target species such as marine mammals and birds that interact with their salmon farms. These data are translated into nature-based categories by way of identifying if any of these non-target species are classified by the International Union for Conservation of Nature (IUCN) as endangered species. In addition, and through certification requirements of the Aquaculture Stewardship Council, wildlife interactions also must be reported to the Global Salmon Initiative, where members' data are aggregated each year (thereby providing something resembling a cumulative account of a sector's nature impacts).
LafargeHolcim Spain [47], an aggregates and cement producer, developed a monitoring system to evaluate restoration processes by studying nature assets/resources based on field samples by cataloguing flora, identifying vegetation, establishing the distribution of birds and insects, assessing the status of biodiversity in the quarry and developing strategies and action plans. Monitoring of activities was undertaken using a biodiversity index developed in collaboration with the WWF and the IUCN's Biodiversity Indicator and Reporting System [48]. While other examples of this kind have been summarized by the EU Business @ Biodiversity Network [49] a dearth of information was observed within the world's largest companies [50].
***Shaping financial system responsiveness***
ASN Bank (as documented in [49]) specializes in sustainability banking products and has developed a biodiversity footprinting tool for financial institutions to allow the impacts of an investment portfolio to be estimated and hotspots in the portfolio identified.
Many banks (for example see NWB Bank and their ‘water bonds’) are issuing bonds to public and private bodies to finance activities that will allow the bond issuer to invest to improve (for example) biodiversity outcomes. This is a relatively niche activity, with the majority of bank funding not having any specific biodiversity screening criteria associated with it. It is, however, anticipated that this might rapidly change.

Several observations can be made based on the examples presented in table 2. First, traceability and geospatial techniques have been used to create links between companies and ecosystems as a pre-condition to both informing company decision-making and providing transparency about activities to external parties. Moreover, this level of linking and transparency includes activities that take place along company value chains, which are by definition outside of the direct control of the entity. Second, the ecological understanding of the data presented has been facilitated by some form of information intermediation. This means that a third party collects, organizes and reproduces information about company actions in a way that makes them more meaningful to others (for example the Ocean Diclosure Project). Third, while there is evidence of some action from shareholders and other providers of finance, given that funders' decisions are not reported publicly it is difficult to be certain of the actual incidence of finance supporting biodiversity outcomes. Without a wholehearted contribution from the finance sector, company action will be severely deterred. Moreover, the focus on individual bank or owner action obscures the fact that the financial system as a whole is currently geared towards nature exploitation and will require radical changes to enable a truly sustainable transformation [51].

## 5. Concluding observations

The aim of this paper was to identify how companies take nature into account in their decision-making practices. The focus was on transnational companies that face a complex landscape of emerging regulation of nature interactions as well as demands for information disclosure about the outcomes of their decision-making. These pressures include both voluntary and binding requirements related to actions and to information disclosure. We have concentrated in large part on the information governance landscape as it provides novel insight for readers of this journal into sources of normativity [52] that may hitherto have been underappreciated. Information translated and interpreted by other actors (including the financial sector) has well-documented effects [36] that provide possibilities for understanding the combined effect of company decision-making. Moreover, advances in traceability and global information systems highlight possibilities for companies to understand impacts and intervene in specific locations [15], thereby countering some of the concerns previously expressed [28,29]. This is not to say that all issues have been resolved as links between companies and their impacts remain temporarily and spatially misaligned; routines, tools and approaches for translating strategy to action are in their early stages and are not always effective; and financial actors are not yet fully aligned with what would be required in an ecologically sustainable global economy. There is much to be done and equally much to be built upon. For example, existing global databases on biodiversity and ecosystems, such as the Global Biodiversity Information Facility (GBIF) or the Ocean Biodiversity Information System (OBIS), were not designed with corporate demands in mind [53]. Yet there is an ever-increasing demand from the private sector to link their activities to credible data on the biosphere (underpinned by traceability technologies). This is an area where organizational scholars and ecologists could collaborate to help translate existing information to support operational changes in companies. We hope that this paper provides insight into points of common interest and concern between these groups of scholars.

## Data Availability

This article has no additional data.
